# Current Practice and Barriers to an Early Antimicrobial Conversion from Intravenous to Oral among Hospitalized Patients at Jimma University Specialized Hospital: Prospective Observational Study

**DOI:** 10.1155/2019/7847354

**Published:** 2019-01-29

**Authors:** Alemseged Beyene Berha, Gizat Molla Kassie

**Affiliations:** ^1^B.Pharm, MSc, Assistant Professor of Clinical Pharmacy, Clinical Pharmacy Unit, Department of Pharmacology and Clinical Pharmacy, School of Pharmacy, College of Health Sciences, Addis Ababa University, Addis Ababa, Ethiopia; ^2^Clinical Pharmacist and Lecturer, Department of Pharmacy, College of Public Health and Medical Sciences, Jimma University, Ethiopia

## Abstract

**Objective:**

The aim of the present study was to explore the current practice and its barriers to an early antimicrobial conversion from intravenous (IV) to oral (PO) therapy among hospitalized patients.

**Method:**

Hospital based prospective observational study was conducted to assess the practice of an early antimicrobial IV to PO conversion and its barriers using medical chart and case-specific physicians' interviews, respectively, from February to September, 2014. Patient charts and medication records were reviewed for appropriateness of IV to PO conversion program every 24hrs using a pretested data collection abstraction format. Independent samples* t*-test was used to compare the duration of therapy and time to clinical stability between converted and nonconverted patients. Two-tailed P values of < 0.05 were regarded as statistically significant.

**Results:**

One hundred forty-two patients were included in the study, of whom two-thirds (67.6%) of the patients were eligible for IV to PO antimicrobial conversion. However, only 20.9% of patients' timely conversion was made. A shorter duration of IV therapy was recorded for converted (2.80±1.87) versus nonconverted patients (8.50±6.32), (P=0.009). The most important barriers of not converting IV to PO in clinically stable patients were presence of comorbidity; clinicians perceived that the patient should always complete IV course of antimicrobials as a standard practice.

**Conclusion:**

Conversion from IV to PO antimicrobials was found to be unnecessarily delayed in a significant proportion of patients hospitalized with moderate to severe infection due to a range of different barriers. Addressing these issues has the potential to reduce inappropriate antimicrobial use and resistance.

## 1. Introduction

In both the community and hospital setting, antibiotics are being commonly prescribed to treat many common infections. Antimicrobial drug uses in hospitals contribute significantly to rising healthcare costs and have been reported to account for up to 25%-50% of a pharmacy department's drug-acquisition budget [1–4]. As defined by the World Health Organization (WHO), the rational use of medicines requires that* “patients receive medications appropriate to their clinical needs, in doses that meet their own requirements, for an adequate time, and at the lowest cost to them and their community”*. The overuse and misuse of antibiotics in hospitals have an influence on therapeutic efficacy, microbial resistance, and cost that make certain picture for the implementation of programs to improve the use of antibiotics in hospitals, particularly in countries with limited resources [5].

One of the strategies to improve rational use of antibiotics is the implementation converting selection of antimicrobials from intravenous (IV) to oral (PO) therapy. IV to PO therapy conversion comprises three types: switch therapy, sequential therapy, and step-down therapy to describe the conversion of IV to PO therapy, using the same or a different compound, as soon as patients are judged to be clinically stable, without losing antimicrobial potency [6]. The term “*antimicrobial conversion”* describes the practice of converting intravenous antimicrobial therapy to an alternative oral formulation; since the 1990s, the IV to PO antibiotic converting programs have been adopted in many countries. Ever since then, many studies have been carried out and had persuasively demonstrated the efficacy, safety, and its cost effect in a health institution [7].

Planning, implementing, and evaluating an IV to PO therapy conversion packages, the ideal medication to include in this program has several characteristics. The formulation of oral dosage form should be well-tolerated when administered orally and have extremely good bioavailability (preferably greater than 80%), and its use should be verified by clinical data. Oral medications are available in multiple dosage forms (e.g., tablets, capsules, and liquids) and dosing at a frequency equivalent to or less than the IV medication gives additional optimal properties and alternative options [6]. Proper identification of patients' diagnosis, medications, and contraindications to oral therapy are all essential aspects for a successful IV to PO therapy conversion program [6]. Patient selection criteria for IV to PO antimicrobial therapy conversion are signs of good clinical response, functioning GI tract as shown by consuming and tolerating scheduled PO medications and oral food intake without signs of nausea, vomiting, or diarrhea [8, 9]. The patients from IV to PO antimicrobial therapy conversion are excluded if they meet any of following criteria: GI obstruction, malabsorption, active GI bleeding, seizure and risk of aspiration, hypotension or shock, and/or intensive care unit (ICU) admission. Patients refusing oral medication as mentioned in chart, immunocompromised patients, or those on antimicrobial therapy with a more severe infection may be excluded from IV to PO conversion program [8–10].

Even though most of the PO antimicrobial agents have excellent bioavailability with similar antimicrobial activity to those parenteral agents, patients who are candidates for conversion were not timely converted to PO antimicrobial therapy [6]. Currently, there is no established document, guidelines, or protocol regarding IV to PO antimicrobial therapy conversion practice in Ethiopia. This may result in an increased cost of medication, hospital acquired infections, work load of clinicians, pharmacists and nurses' duties, hospital stay, and overall healthcare system expenditure.

The escalating cost associated with antimicrobials use and increased antimicrobial resistance have become of increasing concern. A number of strategies have been developed to address these problems. One of the simplest cost savings stewardship initiatives is the implementation to convert selection of antimicrobials from intravenous (IV) to oral (PO) therapy. Many studies have documented a better clinical outcome, reducing lengths of stay in hospital, lesser complications, and cost savings by converting patients from IV to PO therapy [11, 12]. However, only a limited number of researches have described antimicrobial conversion in developing countries [13] and particularly in Ethiopia [14]. Therefore, the aim of this study was to assess the current practice and its barriers to an early antimicrobial conversion from IV to PO therapy in medical and surgical wards of Jimma University Specialized Hospital (JUSH). The findings of the present study are crucial to come up with specific recommendations for the practice of timely antimicrobial conversion from IV to PO therapy, which will have a paramount importance on cost and safer use of antimicrobials.

## 2. Participants and Method

### 2.1. Study Area and Period

The study was conducted in three medical and two surgical wards of JUSH for four months (February–September, 2014). The medical wards have a total of 89 beds with 12 senior physicians (specialists), 28 resident physicians, and 44 nurses. Similarly, the two surgical wards have 78 beds along with 6 senior physicians (specialists), 24 resident physicians, and 41 nurses. In addition, there were various groups of students who were assigned for clinical attachment to both wards.

### 2.2. Study Design

Hospital based prospective observational study was conducted to explore the current practice and its barriers of an early antimicrobial IV to PO conversion using patient chart and case-specific physicians' interviews, respectively, at JUSH. Data regarding the current practice of antimicrobial conversion from IV to PO therapy was collected using a structured checklist.

### 2.3. Inclusion and Exclusion Criteria

All patients were admitted to medical wards for pneumonia and urinary tract infections and to surgical wards for skin and soft tissue infections (e.g., cellulitis, soft tissue laceration, and pyomyositis), and bone and joint infections (e.g., osteomyelitis) during the time of data collection were included to the study.

Patients with serious deep seated infections that often require prolonged IV therapy (e.g., meningitis, endocarditis, deep abscess, cystic fibrosis, and infection of a prosthetic device) were excluded. In addition, patients with recognized surgical prophylactic schemes lasting less than 24 hours, neutropenia (leukocyte count <0.5 x 10^9^/L), hospital acquired pneumonia, and long treatment duration (> two months) with unsettled working diagnosis were excluded.

### 2.4. Data Collection

Data was collected by five trained nurses working in the wards. Patient data extraction tool (in English) had six distinct parts. To maintain the quality of the data, a checklist was prepared and pretested for its completeness for coverage of critical domains and wording clarity on randomly selected patients' record.

The first part collected demographic data including age, sex, educational level, residence, monthly income, and type of ward that the patient was admitted. The second section collected information regarding diagnosis and prescribed medications; the third section comprised patient inclusion and exclusion criteria for converting to oral antimicrobials, adapted from Laing et al. [15] and Senn et al. [5]. The forth section asked physicians to respond to the barriers of an early antimicrobial IV to PO conversion for clinically stable patients from list of possible reasons or open ended question. The fifth section comprised the duration of antimicrobial therapy, clinical stability, and hospital stay. The sixth section also consisted of vital sign sheet together with route of antimicrobial administration

Within 24 hrs upon admission, data of all patients and their routes of antimicrobial (IV/ orally) were assessed and recorded. Criteria for clinical stability in hospitalized patients were defined as normalization of vital signs such as heart rate <100 beats per minute; respiratory rate, <25 breaths per minute; temperature, < 37.8°C; oxygen saturation, >92% devoid of further administration of oxygen; normal blood pressure without the demand for saline infusion or vasopressive medication; and normal mental status that appeared following the onset of infection [16, 17]. If patients were able to swallow and were relived nausea and/or vomiting they were noticeable as* “able to take oral medication”*. The vital signs not documented in the medical chart were recorded by the data collector nurses. Recording of clinical data continued after a patient was converted to oral antibiotics for at least 72 hrs.

### 2.5. Data Processing and Analysis

The collected data was cleaned, categorized, and coded and was entered in Epi info version 7. The data were entered and analyzed using SPSS for windows version 21.0. Two-tailed P values of < 0.05 were considered statistically significant.

Independent samples* t*-test was used to compare the duration of therapy, time to clinical stability, observation period after conversion, length of hospital stay, and total antibiotic acquisition costs between converted and nonconverted patients.

## 3. Results

### 3.1. Sociodemographic Data

A total of 164 patients who had been admitted in JUSH with a disease of community acquired pneumonia (CAP), urinary tract infection (UTI), and skin and soft tissue infections (SSTI) were included in the study. Of them, twenty-two patients were excluded due to two patients who had a long duration of antibiotics treatment (> two months) with difficulty of diagnosis conformation. Seven patients had missing data and thirteen patients died before intravenous to oral conversion. Of these, 142 patients were analyzed (Table 1).

The mean age of patients was 39.45 (± 16.44) and 72 (50.7%) females. The four most common admission diagnoses were CAP (67.6%), SSTI (9.9%), CAP plus UTI (9.9%), and UTI (8.5%). One hundred two (71.8%) patients had one or more comorbid diseases; the top three chronic diseases before hospital admission were cardiovascular disease (54.9%), tuberculosis (25.5%), and diabetes mellitus (11.8%) (see Table 2).

### 3.2. Barriers for Early IV to PO Conversion

**Figure 1:**
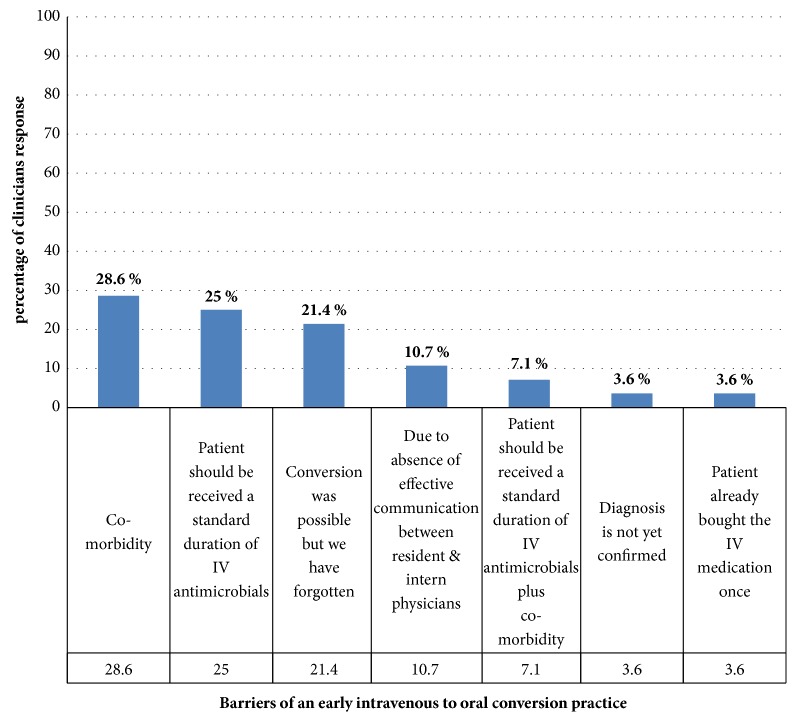
Physicians' response about barriers of an early antimicrobial IV to PO conversion practice at Jimma University Specialized Hospital, South West Ethiopia, February–September 2014 (n=28).

By the use of patients-specific interviews, we had asked a total of 28 attending physicians about the barriers of an early antimicrobials IV to PO conversion in clinically stable patients. Of them, 8 (28.6%) clinicians reported that due to the presence of comorbidities. In addition, one resident physician responded that the patient already bought IV medications once (see Figure 1).

### 3.3. Incidence of Antimicrobial IV to PO Conversion

**Figure 2:**
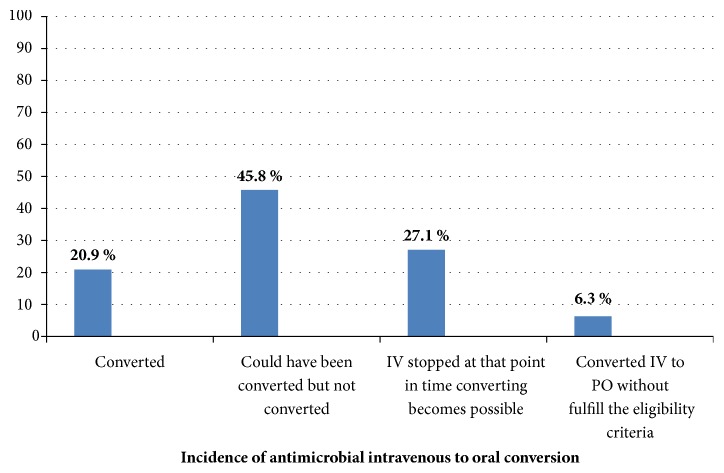
The percentage of patients in the incidence of antimicrobial IV to PO conversion at Jimma University Specialized Hospital, South West Ethiopia, February–September 2014 (n=96).

From a total of 142 patients, 96 (67.6%) who started IV antimicrobials were eligible for intravenous to oral antimicrobial conversion. However, from eligible subjects only 20 (20.9%) patients were timely converted, 44 (45.8%) patients could have been converted but not converted, 26 (27.1%) patients were IV stopped at point that converting become possible, and 6 (6.3%) of patients were converted without fulfilling eligibility criteria (see Figure 2).

### 3.4. Antimicrobials Prescribed, Time to Clinical Stability, Observation Period after Conversion, and Length of Hospital Stay

The total of 106 (74.6%) records of patients receiving ceftriaxone was reviewed. Out of these, each of four patients received ampicillin and cloxacillin plus metronidazole. Also, cloxacillin plus chloramphenicol was prescribed to 16 (11.3%) patients and also 4 patients received ceftazidime. The most common oral antimicrobial prescribed for conversion program was amoxicillin with or without clavulanic acid 51.8% (28/54) and chloramphenicol plus cloxacillin 29.6% (16/54). The time taken for a patient to reach clinical stability was 6.04±3.25 (n=136). Converted patients commonly had a shorter time to clinical stability (1.03–5.77 days) than nonconverted patients (3.37–7.45 days) (P=0.020).

Converted patients were observed in hospital after the initiation of oral therapy for a mean of 5.18 days; as a result converted patients (9.0±5.23) spent short duration in hospital than nonconverted patients (13.45±5.48) (P=0.039).

### 3.5. Duration of IV Antimicrobial Therapy

A shorter duration of IV therapy was recorded for converted (3.30 ± 2.26) versus nonconverted patients (8.64 ± 2.70) (P=0.009). Also, the duration of IV therapy received by nonconverted patients after clinical stability was 1.14–5.32 days (3.23± 2.09). The number of IV antimicrobial prescriptions for which duration of treatment was specified by physicians was 87 (see Table 3).

### 3.6. Cost Implications

Regarding the cost of medication, a total amount of Ethiopia birr 3074.84 (n=20) was saved for converted patients and a further Ethiopia birr 4080.06 (n= 44) could have been saved if nonconverted patients had also been converted.

### 3.7. Combined Use of IV Antibiotics and Oral Medications

All CAP patients, while being admitted in ward, immediately took IV ceftriaxone or ceftazidime with oral doxycycline even if the patient was not candidate to oral medication due to the absence of IV first line drug that could be substituted by oral doxycycline. Among eligibility criteria for IV to PO conversion, the patients able to tolerate oral medications were important. In addition, it was valuable to document the number of oral prescribed drugs for and received by the patients on top of IV antimicrobial therapy. The number of oral drug prescriptions issued to inpatients beside IV antimicrobial therapy was 96. Furthermore, the fact that near half of patients 45.07% ( 64/142) received oral drugs along with IV antimicrobial therapy indicated that more than half of patients had gastrointestinal absorption problems or PO medication might not be required. However, all CAP patients were taking oral doxycycline from the beginning.

## 4. Discussion

To the best of our knowledge, this is the second study carried out in Africa with the greatest challenge to the effective treatment of infectious disease due to antimicrobial resistance and escalating costs of antimicrobials with the suitability of converting antibiotic therapy from the intravenous to the oral route.

An appropriate antimicrobial utilization is a cornerstone for the containment of antimicrobial resistance and good clinical and economic outcomes. Antibiotic resistance is one of the most urgent public health problems of increasing magnitude and probing effective way out to address this difficulty. With the aim of decreasing the selective pressure of antibiotics, it is useful to make sure that when antibiotics are used, they are used properly by tackling unnecessary use of IV antibiotics; smooth and timely conversion to oral antibiotics package could reduce hospital acquired disease complication from IV antibiotic delivery, reduce length of stay in the hospital, and reduce overall use of antibiotics and associated costs of antibiotics. This study was therefore designed to assess the current practice and its barriers to an early antimicrobial IV to PO conversion in hospitalized patients at JUSH.

The higher proportion of admission diagnosis was CAP (67.6%) and the most frequent chronic disease present before admission was cardiovascular disease (54.9%). Those findings are consistent with the previous study in South Africa 2011 [18]. Conversely, Kari E. Kurtzhalts et al., 2015, studied 174 patients being treated for community acquired and healthcare-associated pneumonia and found a comorbid disease such as chronic obstructive pulmonary disease 43.68% (76), diabetes 32.76% (57), and heart failure 27.01% (47), respectively [19]. The proportion of converted patients 20.8% (20/96) in this study was higher from that reported by Van Niekerk et al. 2012 (13%) [18]. Published studies enrolling patients with various infectious diseases in several European countries and the USA suggest that ~30% to more than 50% of patients could be switched from IV to PO antibiotic therapy [20–24]. These rates are lower than those observed within this study hospital (20.8%). So, considerations of possible conversions are low in a resource limited country like Ethiopia.

The mean time to clinical stability in patients hospitalized (6.04 ± 3.25 days) was slightly increased from the previous studies in preimplementation phase (4.7±2.5) by Van Niekerk et al. 2012 [18] and that also revealed 2.0–4.0 days as the suitable time for IV therapy to be reevaluated [5, 25, 26]. The increase in time to clinical stability might raise the inquiry of whether antimicrobials were given for either too long (unnecessary use) or too short (risk of relapse) period. However, the decision to conversion was left to the attending physicians and, thus, it was assumed that patients were appropriately converted according to the discretion of the physician with the help of the conversion criteria. After conversion, most of the converted patients stayed in hospital. This could be linked to the high incidence of comorbid conditions, such as cardiovascular disease, tuberculosis, and diabetes mellitus or socioeconomic factors (a lack of money, absence of reliable family members, and/or the absence of equipped facility for continued care). Other reasons could include (i) physicians reluctant to discharging patients; (ii) patients not assessed on a daily basis; (iii) physicians waiting for further diagnostic workup, similar to previous studies [15, 25]; (iv) the influence of consumerist dynamics within the doctor–patient relationship and resultant practices to avoid conflict or even litigation; and (v) ripple effects of hierarchical structures within medical team in terms of juniors making timely changes to antibiotic prescriptions [27].

The median length of hospitalization for patients in this study (11 days) (Table 3) was slightly higher to the median length of hospitalization reported by Van Niekerk et al. 2012, (9.2 days) at preimplementation phase [18]; this could be most of patients in this study having a severe infection as compared to the previous study. The number of prescriptions for which IV antimicrobials duration was specified by physicians in this study 7(n=174) was decreased from the prior work by Van Niekerk et al. 2012, 40 (n=204) at preimplementation phase [18]. The possible reasons for this difference could be differences in patient demographics and less availability of alternative medication in the current study hospital. The median duration of IV antimicrobial in the present study (7 days) was close to the previous study by Mertz et al. [25] (6 days) at control phase. The mean time of IV therapy after clinical stability for nonconverted patients in this study (3.23 ± 2.09 days) was consistent with the prior work by Van Niekerk et al. [18] ( 3.8 ± 2.4). In the current study, the considerable decrease in the duration of IV therapy led to substantial drug-acquisition cost savings of up to 3074.84 birr (n=20) or $ 111.76 (n=20) in the converted patients. This result was similar to finding in the prior work by Van Niekerk et al. [18], $ 113.63 (n=19) at preimplementation phase.

In this study, all CAP patients at the time of admission were taking oral doxycycline along with IV ceftriaxone or ceftazidime, even if the patient was not candidate for oral medication, due to the absence of first line IV medication that could substitute oral doxycycline. A recent survey discovered that pharmacists in the USA regard gastrointestinal functionality as one of the most important criteria for converting from IV to oral therapy [28]. In the current study, near half of patients 45.07% (64/142) received oral drugs while on IV antimicrobial therapy this indicated that more than half of patients had gastrointestinal absorption problems or it might not require PO medication. However, all community acquired pneumonia patients were taking oral doxycycline from the beginning. This implies that since 45.0% of patients received oral medication while on IV antimicrobial therapy, the number of patients that can take oral medication could have been even higher than the converted patients in current study (20.8%) and other study reports [29]. This study has taken into account that certain clinical conditions, such as infective endocarditis, meningitis, deep abscess, cystic fibrosis, infection of a prosthetic device, and neutropenia, require IV therapy even though the patient can take oral medication (due to the pharmacokinetic/pharmacodynamic criteria of the antimicrobial).

To know the perceived barriers to an early antimicrobial IV to PO conversion strategy from the treating physicians, they were asked case-specific query using structured checklist and responded with the following reasons: patients with the presence of comorbid disease 28.6% (8/28), the patients that should receive standard duration of IV antimicrobials (IV antibiotics were perceived as more potent and having significant mythical qualities) 25.0% (7/28), and those who forget to convert from IV to PO medication 21.4% (6/28). These findings were consistent with the study done by Warburton et al., 2014 [30]. Conversely, those findings were inconsistent with the theory response (physician specific questionnaire) about reasons of continuing IV therapy comprising clinical instability 97.2% (106/109), uncertainty about gastrointestinal function 84.4 (92/109), and uncertainty as to whether the oral alternatives achieve effective tissue level 78.0% (85/109). It is therefore likely that the majority of the barriers identified in this study could be reduced by means of an educational intervention [31, 32]. These results suggest important policy implications for Ethiopia that should be further investigated in larger patient samples across the country.

## 5. Conclusion

Conversion from intravenous to oral antimicrobials was found to be unnecessarily delayed in a significant proportion of patients hospitalized with moderate to severe infection due to a range of different barriers. Addressing these issues has the potential to reduce inappropriate antimicrobial use and resistance.

In effect, the preliminary finding of this research opens a road map for further exploration of practice and perceived barriers for effective implementation of intravenous to oral conversion at hospitals. Clinical pharmacist and treating physicians should come together to work hand-in-hand to improve the practice of antimicrobials therapy. As the impact of intravenous to oral conversion on the incidence of IV device-related infections and rate of relapse and detailed cost containment issue were not assessed in the current study, it is also clear that further studies are necessary for investigating these aspects.

## Figures and Tables

**Table 1:** Sociodemographic characteristics of patients at Jimma University Specialized Hospital, South West Ethiopia, February–September, 2014 (n=142).

**Characteristics**	**Respondents**
	**N (**%**)**
**Demographic data **	
Subjects	142(100)
Age in years	39.45 ± 16.44
Gender	
Male	70(49.3)
Females	72(50.7)
Educational level	
Illiterate	78(54.9)
Primary school	36(25.4)
Secondary school	18(12.7)
College and above	10(7.0)
Residence	
Urban	66(46.5)
Rural	76(53.5)
Monthly income	
<501	36(25.4)
501-1000	84(59.2)
1001-2000	18(12.7)
Above 2000	4(2.8)

**Table 2:** Pattern of diseases and prescribed drugs for intravenous to oral converting practice at Jimma University Specialized Hospital, South West Ethiopia, February–September, 2014 (n=142 ).

**Characteristics**	**Respondents**
	**N (**%**)**
**Diagnosis for antimicrobial therapy (n= 142) **	
Community acquired Pneumonia(CAP)	96(67.6)
Skin and soft tissue infection	14(9.9)
Community acquired pneumonia + UTI	14(9.9)
Urinary tract infection (UTI)	12(8.5)
Bone and joint infection	4(2.8)
Urinary tract infection + bone and joint infection	2(1.4)

**Co-morbidity (n=102)**	
Cardio vascular disease (CVD)	56(54.9)
Tuberculosis	26(25.5)
Diabetes mellitus	12(11.8)
Human immunodeficiency virus (HIV)	2(2.0)
>1 Co-morbidity	6(6.0)

**Patients were receiving intravenous antimicrobials (n=142)**	
Ceftriaxone	106(74.6)
Chloramphenicol + Cloxacillin	16(11.3)
Ceftazidime	4(2.8)
Ampicillin + Ceftriaxone	4(2.8)
Ceftriaxone + Cloxacillin + Metronidazole	4(2.8)
Cloxacillin + Ceftriaxone	2(1.4)
Gentamicin +Ceftriaxone	2(1.4)

**Table 3:** Intravenous to oral antimicrobial therapy conversion outcomes at Jimma University Specialized Hospital, South West Ethiopia, February–September, 2014.

Variable	Implementation
**Duration of IV therapy **	
** all patients **	
** mean± SD **	7.66 ± 3.25 (n=142)
** median; range**	7; 0 – 15
** converted patients **	
** mean± SD**	3.30 ± 2.26 (n=20)
** non-converted patients **	
** mean± SD**	8.64 ± 2.70 (n=44)

**Time to clinical stability (days ) **	
** all patients **	
** mean± SD**	6.04± 3.25 (n=136)
** median; range**	5;0 – 16
** converted patients **	
** mean± SD**	3.40± 2.37 (n=20)
** non-converted patients **	
** mean± SD**	8.69± 3.73(n=44)

**Observation period after conversion (days )**	
** mean± SD**	5.18± 4.60 (n=28)
** median; range**	4.5;0 – 15

**Duration of IV therapy after clinical stability ** ** for non-converted patients (days) **	
** mean ±SD**	3.23± 2.09 (n=44)
** median; range**	3; 0 – 8

** Number of IV antimicrobial prescriptions for which duration was specified (n=total number of prescriptions)**	7(n=174)

**Length of hospital stay (days)**	
** all patients **	
** mean± SD**	13.42 ± 7.89(n=142)
** median; range**	11; 1 – 44
** converted patients **	
** mean± SD**	9.0 ± 5.23 (n=20)
** non-converted patients **	
** mean± SD**	13.45 ± 5.48 (n=44)

** Total antibiotic acquisition costs (birr)**	24495.04
** Cost saving analysis**	
** Total amount saved**	
** Converted groups**	3074.84 (n=20)
** Non-converted groups **	4080.06 (n= 44)

## Data Availability

The derived data used to support the findings of this study are available from the corresponding author upon request.

## Abbreviations

CAP: Community acquired pneumonia
COPD: Chronic obstructive pulmonary disease
IV to PO: Intravenous to oral
IV: Intravenous
JUSH: Jimma University Specialized Hospital
PO: Oral
UTI: Urinary tract infections.

## References

[1] Pablos A. I., Escobar I., Albiñana S., Serrano O., Ferrari J. M., de Tejada A. H. (2005). Evaluation of an antibiotic intravenous to oral sequential therapy program. *Pharmacoepidemiology and Drug Safety*.

[2] Polk R. E. (1987). The role of the infectious diseases physician in monitoring antimicrobial use: a pharmacy perspective. *Journal of Urban Health : Bulletin of the New York Academy of Medicine*.

[3] Berman J. R., Zaran F. K., Rybak M. J. (1992). Pharmacy-based antimicrobial-monitoring service. *American Journal of Health-System Pharmacy*.

[4] Powers D. A. (1986). Antimicrobial surveillance in a VAMC teaching hospital - Resulting cost avoidance. *Drug Intelligence & Clinical Pharmacy*.

[5] Senn L., Burnand B., Francioli P., Zanetti G. (2004). Improving appropriateness of antibiotic therapy: Randomized trial of an intervention to foster reassessment of prescription after 3 days. *Journal of Antimicrobial Chemotherapy*.

[6] Kuper K. M., Murdaugh L. B. (2008). Intravenous to Oral Therapy Conversion. *Competence Assessment Tools for Health-System Pharmacies 4edn*.

[7] Wetzstein G. A. (2000). Intravenous to oral (IV:PO) anti-infective conversion therapy. *Cancer Control*.

[8] Glemaud I. (2000). Use of a physician order entry system to identify opportunities for intravenous to oral levofloxacin conversion. *American Journal of Health-System Pharmacy*.

[9] Hunter K. A., Dormaier G. K. (1995). Pharmacist-managed intravenous to oral step-down program. *Clinical Therapeutics*.

[10] Mettler J., Simcock M., Sendi P. (2007). Empirical use of antibiotics and adjustment of empirical antibiotic therapies in a university hospital: A prospective observational study. *BMC Infectious Diseases*.

[11] Przybylski K. G., Rybak M. J., Martin P. R. (1997). A pharmacist-initiated program of intravenous to oral antibiotic conversion. *Pharmacotherapy*.

[12] Craig W. A., Andes D. R. (1995). Parenteral versus oral antibiotic therapy. *Medical Clinics of North America*.

[13] Engel M. F., Postma D. F., Hulscher M. E. J. L. (2013). Barriers to an early switch from intravenous to oral antibiotic therapy in hospitalised patients with CAP. *European Respiratory Journal*.

[14] Mandell L. A., Bergeron M. G., Gribble M. J. (1995). Sequential Antibiotic Therapy: Effective Cost Management and Patient Care. *Canadian Journal of Infectious Diseases & Medical Microbiology*.

[15] Laing R. B. S., Mackenzie A. R., Shaw H., Gould I. M., Douglas J. G. (1998). The effect of intravenous-to-oral switch guidelines on the use of parenteral antimicrobials in medical wards. *Journal of Antimicrobial Chemotherapy*.

[16] Halm E. A., Fine M. J., Marrie T. J. (1998). Time to clinical stability in patients hospitalized with community- acquired pneumonia: Implications for practice guidelines. *Journal of the American Medical Association*.

[17] Menéndez R., Torres A., Rodríguez De Castro F. (2004). Reaching stability in community-acquired pneumonia: The effects of the severity of disease, treatment, and the characteristics of patients. *Clinical Infectious Diseases*.

[18] van Niekerk A. C., Venter D. J. L., Boschmans S.-A. (2012). Implementation of intravenous to oral antibiotic switch therapy guidelines in the general medical wards of a tertiary-level hospital in South Africa. *Journal of Antimicrobial Chemotherapy*.

[19] Kurtzhalts K., Sellick J., Ruh C., Mergenhagen K. (2015). Impact of Antimicrobial Stewardship on Outcomes in Hospitalized Veterans With Pneumonia. *Clinical Therapeutics*.

[20] Desai M., Franklin B. D., Holmes A. H. (2006). A new approach to treatment of resistant gram-positive infections: Potential impact of targeted IV to oral switch on lenght of stay. *BMC Infectious Diseases*.

[21] Parodi S., Rhew D. C., Goetz M. B. (2003). Early switch and early discharge opportunities in intravenous vancomycin treatment of suspected methicillin-resistant staphylococcal species infections.. *Journal of managed care pharmacy : JMCP*.

[22] Buyle F. M., Metz-Gercek S., Mechtler R. (2012). Prospective multicentre feasibility study of a quality of care indicator for intravenous to oral switch therapy with highly bioavailable antibiotics. *Journal of Antimicrobial Chemotherapy*.

[23] Sevinç F., Prins J. M., Koopmans R. P. (1999). Early switch from intravenous to oral antibiotics: Guidelines and implementation in a large teaching hospital. *Journal of Antimicrobial Chemotherapy*.

[24] Dryden M., Saeed K., Townsend R. (2012). Antibiotic stewardship and early discharge from hospital: Impact of a structured approach to antimicrobial management. *Journal of Antimicrobial Chemotherapy*.

[25] Mertz D., Koller M., Haller P. (2009). Outcomes of early switching from intravenous to oral antibiotics on medical wards. *Journal of Antimicrobial Chemotherapy*.

[26] Athanassa Z., Makris G., Dimopoulos G., Falagas M. E. (2008). Early switch to oral treatment in patients with moderate to severe community-acquired pneumonia: A meta-analysis. *Drugs*.

[27] Broom J., Broom A., Adams K., Plage S. (2016). What prevents the intravenous to oral antibiotic switch? A qualitative study of hospital doctors' accounts of what influences their clinical practice. *Journal of Antimicrobial Chemotherapy*.

[28] Banko H., Goldwater S. H., Adams E. (2009). Smoothing the path for intravenous (IV) to oral (PO) conversion: Where have we come in 11 years?. *Hospital Pharmacy Journal*.

[29] Houfi A. E., Javed N., Solem C. T. (2015). Early-switch/early-discharge opportunities for hospitalized patients with methicillin-resistant staphylococcus aureus complicated skin and soft tissue infections: Proof of concept in the united arab emirates. *Infection and Drug Resistance*.

[30] Warburton J., Hodson K., James D. (2014). Antibiotic intravenous-to-oral switch guidelines: Barriers to adherence and possible solutions. *International Journal of Pharmacy Practice*.

[31] Schouten J. A., Hulscher M. E. J. L., Natsch S., Kullberg B.-J., Van Der Meer J. W. M., Grol R. P. T. M. (2007). Barriers to optimal antibiotic use for community-acquired pneumonia at hospitals: A qualitative study. *Quality & Safety in Health Care*.

[32] Kamarudin G., Penm J., Chaar B., Moles R. (2013). Educational interventions to improve prescribing competency: A systematic review. *BMJ Open*.

